# Necessary Harmony Between Anesthesia and Neurosurgery During Extracranial–Intracranial Bypass: A Review of Neuroanesthesia Strategies and Perioperative Insights

**DOI:** 10.3390/neurosci6040096

**Published:** 2025-10-01

**Authors:** Vincent Bargnes, Wesam Andraous, Nicholas Bitonti, Zhaosheng Jin, Sofia Geralemou

**Affiliations:** 1 Department of Anesthesiology, Stony Brook University Hospital, Stony Brook, NY 11794, USA; vincent.bargnes@stonybrookmedicine.edu (V.B.III); wesam.andraous@stonybrookmedicine.edu (W.A.); sofia.geralemou@stonybrookmedicine.edu (S.G.); 2 Renaissance School of Medicine, Stony Brook University, Stony Brook, NY 11794, USA; nicholas.bitonti@stonybrookmedicine.edu

**Keywords:** extracranial–intracranial bypass, EC–IC, vascular neurosurgery, cerebral revascularization, literature review

## Abstract

The extracranial–intracranial (EC–IC) bypass is a complex neurosurgical procedure performed for cerebral flow augmentation or flow replacement. Anesthetic management of these patients poses significant challenges due to the delicate balance required to maintain cerebral perfusion, often complicated by extensive cardiovascular comorbidities. Despite the complexity of these cases, current literature offers limited guidance on optimal anesthetic strategies. At our high-volume academic institution, we developed a standardized multimodal anesthetic protocol aimed at achieving intraoperative hemodynamic stability and facilitating timely postoperative emergence. A dedicated team of neuroanesthesiologists manages these cases in constant communication with the surgical team, ensuring real-time adjustments aligned with surgical needs and patient physiology. Our experience highlights the importance of individualized anesthetic planning and interdisciplinary coordination. Given the scarcity of published data and the specialized nature of EC–IC bypass procedures, we believe our institutional approach may serve as a useful reference for other centers, particularly those with limited exposure to this complex patient population, and lay the foundation for future prospective trials on optimal anesthetic care for this patient population.

## 1. Introduction

Extracranial–intracranial (EC–IC) bypass is a microsurgical revascularization procedure used to augment or replace cerebral blood flow in patients at risk of ischemia. First proposed by C. Miller Fisher in 1951 and performed by Yasargil in the late 1960s, the technique typically involves an anastomosis between the superficial temporal artery and the middle cerebral artery [1,2]. EC–IC bypass is primarily indicated for Moyamoya disease, intracranial atherosclerosis, and complex aneurysms requiring vessel sacrifice [3,4,5].

The procedure is designed to augment flow in hypoperfused territories or replace flow when major arteries are compromised. Despite growing clinical use, anesthetic management remains technically demanding and underrepresented in the literature. Evidence suggests that higher volume EC–IC bypass centers have improved outcomes as entire perioperative teams can retain and hone specialized expertise [6]. 

Anesthetic challenges include maintaining stable cerebral perfusion during temporary vessel occlusion, avoiding excessive vasoconstriction during graft harvesting, and preventing significant hemodynamic variability that could compromise graft patency [7]. Advanced neuromonitoring—such as electroencephalograms, somatosensory evoked potentials, Doppler ultrasound, and indocyanine green angiography—can help guide intraoperative decisions [8].

Despite growing use of EC–IC bypass, literature on anesthetic management remains sparse. This review highlights key anesthetic considerations based on available evidence and clinical experience, with the aim of encouraging further research and supporting anesthesiologists in optimizing care during these complex surgical procedures.

## 2. Indications for Cerebral Bypass Surgery

EC–IC bypass is most often considered in select aneurysms where traditional vascular interventions like endovascular coiling or microsurgical clipping are unfeasible or too high a risk. While early studies from the 1980s did not support a benefit in stroke prevention, advances in imaging and surgical techniques have changed the role of bypass in modern medical practice [9].

Today, the most recognized indication for EC–IC bypass includes giant or complex aneurysms, particularly involving the internal carotid artery (ICA), where parent vessel occlusion is part of the treatment strategy. In such cases, bypass is required to adequately perfuse distal tissue [10,11]. Moyamoya disease (MMD) is another well-established indication, as it is characterized by progressive bilateral ICA stenosis or occlusion, resulting in hypoperfusion. In this population, EC–IC bypass and the resultant cerebral blood flow augmentation can be either prophylactic or therapeutic [12].

Additionally, patients with intracranial atherosclerotic disease who remain symptomatic despite maximal medical therapy may benefit from revascularization. Advanced perfusion imaging and balloon test occlusion are able to assist in identifying those most likely to achieve hemodynamic benefit [13]. EC–IC bypass is also occasionally employed prophylactically in cases where major vessels must be sacrificed, such as during skull base tumor resections or treatment of vascular neoplasms that involve the ICA. [14]. In these scenarios, it is done to avoid infarction preventively [15,16]. Broadly, EC–IC bypass can be categorized into prophylactic interventions for high-risk but asymptomatic vascular compromise and therapeutic interventions for progressive ischemia refractory to conservative management.

## 3. Technical Considerations

### 3.1. High and Low Flow Bypass

EC–IC bypass procedures can be divided into high-flow and low-flow categories. High-flow bypasses consist of radial artery or saphenous vein grafts and deliver blood flow rates that can surpass 100 mL/min [17]. Compared to low-flow bypasses, they are better suited for cases involving parent vessel occlusion to treat complex aneurysms or tumors. These grafts connect the external carotid artery to a major intracranial vessel, like the M1 segment of the middle cerebral artery (MCA). High-flow procedures often require the grafts to be positioned in the direction of normal venous flow to prevent valve interference and need to be carefully planned to avoid other issues like graft kinking, turbulent flow, or thrombosis [18,19,20].

In contrast, low-flow bypasses usually provide 15–50 mL/min of flow, which is still significant enough to improve perfusion. These bypasses typically involve anastomoses between the superficial temporal artery and MCA cortical branch. They can also involve connection of the superficial temporal artery to the posterior or anterior cerebral artery. Low-flow bypass can also be between the occipital artery and the posterior cerebral artery. These procedures are typically better suited for smaller ischemic territories or in patients with Moyamoya disease. Low-flow procedures are favored for their shorter operative times and lower risk profile [21,22].

Deciding between high-flow and low-flow approaches in vascular surgery is not solely determined by the disease process, but also by detailed anatomical assessments. Key factors to consider include the diameter and course of both the donor and recipient vessels and the quality of collateral pathways like the circle of Willis. Preoperative imaging, like CT perfusion, MR angiography, and digital subtraction angiography, is also a key enabling factor in tailoring the bypass type to the patient’s specific anatomy [23].

Successful graft placement and vessel selection are not the only elements that determine if proper cerebral perfusion can be achieved. Physiological factors such as blood pressure, vascular resistance, and cerebrovascular reserve also influence outcomes. Even when the graft is patent, the benefit of the bypass depends on the pressure gradient across the graft, recipient artery capacity, and the microvascular integrity of downstream vessels. Postoperative imaging studies often reveal variable flow dynamics across different territories, influenced by the flow from native vessels and the adaptability of distal arterioles [24].

High-flow bypasses do not always lead to improved perfusion if there is resistance at the recipient site or autoregulatory vasoconstriction occurs in response to over-perfusion. Conversely, despite their limited capacity, low-flow bypasses may improve cerebrovascular reactivity by stabilizing perfusion pressure in hypoperfused regions [22].

Collateral circulation also plays a role in perfusion outcomes. Patients with stronger leptomeningeal collaterals or an intact circle of Willis can demonstrate adequate perfusion with smaller bypass flows, while individuals with poorer collateralization may need stronger revascularization strategies [21,22].

### 3.2. Determinant of Cerebral Perfusion

Despite comprising only about 2% of total body weight, the brain consumes nearly 20% of the body’s oxygen, underscoring its status as one of the most metabolically active organs. This high oxygen demand necessitates a robust autoregulatory system that maintains consistent cerebral blood flow (CBF) across a wide range of physiological conditions [25]. This mechanism has evolved to protect the brain from ischemia, as even brief interruptions in perfusion can result in neuronal injury or death. With the evolution of upright posture and higher-level cognitive functions in humans, the need for stable cerebral perfusion has become critical, forming the physiological foundation for the extracranial-to-EC–IC bypass procedure. A thorough understanding of cerebral autoregulation is vital when designing a neuroanesthetic plan for patients undergoing EC–IC bypass and is equally important for perioperative care teams involved in managing these complex cases.

#### 3.2.1. Mean Arterial Pressure

Cerebral perfusion is governed by the pressure gradient between the systemic arterial circulation and the intracranial arterial circulation, known as cerebral perfusion pressure (CPP) [26]. While most body tissues rely on mean arterial pressure (MAP)—the average pressure generated by the left ventricle during the cardiac cycle—for perfusion, the brain operates under additional constraints due to its enclosure within the rigid skull. This fixed cranial vault introduces intracranial pressure (ICP) as a counterforce that must be overcome for blood to adequately perfuse brain tissue. Consequently, CPP is defined as the difference between MAP and ICP (CPP = MAP − ICP). In a healthy individual, MAP typically ranges from 70 to 100 mmHg, and ICP from 5 to 15 mmHg, resulting in a normal CPP of approximately 60 to 80 mmHg. In pathological conditions such as traumatic brain injury or intracranial hemorrhage, elevated ICP—often due to accumulating blood or edema—can significantly reduce CPP, thereby impairing CBF and increasing the risk of ischemia.

The first layer of cerebral perfusion autoregulation occurs early in the cerebral vasculature, where major arteries respond to changes in MAP through vasoconstriction or vasodilation. At higher MAPs, cerebral vessels constrict to limit excessive blood flow and protect delicate neural tissues from overperfusion. Conversely, at lower MAPs, these vessels dilate to maintain adequate perfusion to brain tissue. This autoregulatory response follows Poiseuille’s Law, which states that small changes in vessel radius can result in large changes in flow—mathematically, flow is proportional to the fourth power of the radius [27]. The underlying mechanism is thought to be primarily myogenic: increased CPP stretches vascular smooth muscle, triggering a cascade involving myosin light chain kinase (MLCK) that results in vasoconstriction [28].

This myogenic autoregulation typically maintains stable CBF within a MAP range of 50–150 mmHg, although more recent studies suggest a narrower range of 70–150 mmHg [29,30]. Notably, the autoregulatory threshold is not fixed across the population and is likely shifted in individuals with select conditions such as chronic hypertension [31]. In these patients, repeated exposure to elevated MAP leads to adaptive changes, shifting the autoregulatory curve to accommodate higher blood pressures and prevent overperfusion damage from consistently elevated MAPs, rereferred to as rightward shift in MAP on the x-axis in relationship to CBF on the y-axis [32]. As a result, a MAP of 70 mmHg—which may be acceptable in normotensive individuals—could fall below the lower limit of autoregulation in patients with chronic hypertension, risking cerebral hypoperfusion. In the context of neuroanesthesiology, precise, individualized blood pressure management is essential. Intraoperative blood pressure must be carefully controlled with vasopressors or vasodilators as needed to preserve cerebral perfusion while maintaining an optimal neurosurgical field.

#### 3.2.2. Arterial Blood Gases

In addition to myogenic mechanisms driven by changes in MAP, CBF is also regulated by arterial blood gases. Elevated partial pressure of carbon dioxide in cerebral arteries (PaCO_2_) leads to vasodilation of cerebral vessels, thereby increasing CBF. Conversely, reduced PaCO_2_ causes vasoconstriction, resulting in decreased CBF. Although the exact mechanisms remain partially understood, this regulation may involve changes in cerebrospinal fluid (CSF) pH or direct effects on cerebral vascular endothelium [33]. The impact of PaCO_2_ is significant: for every 1 mmHg increase above 40 mmHg, CBF increases by approximately 4%, with a corresponding decrease in flow during hypocapnia [34]. Partial pressure of oxygen (PaO_2_) also influences cerebral autoregulation, though to a lesser extent than PaCO_2_. Vasodilation typically occurs only in the setting of significant hypoxemia, usually when PaO_2_ drops below 50 mmHg [35].

Given their profound influence on cerebral perfusion, blood gases are central to neuroanesthetic management. Invasive arterial pressure monitoring and mechanical ventilation enable precise control of PaCO_2_ and PaO_2_, supported by point-of-care blood gas analysis. Effective regulation of arterial blood gases serves as an important component of cerebral autoregulation and contributes to optimal perioperative neurosurgical outcomes.

#### 3.2.3. Cerebral Metabolic Rate of Oxygen

Cerebral blood flow (CBF) is closely coupled to the cerebral metabolic rate of oxygen consumption (CMRO_2_). As brain tissue becomes more metabolically active, it produces vasoactive byproducts—such as adenosine, nitric oxide (NO), and various ions including hydrogen (H^+^), potassium (K^+^), calcium (Ca^2+^), and lactate—that promote local vasodilation [36]. This neurovascular coupling ensures that increases in neuronal activity are met with corresponding increases in perfusion to meet metabolic demands.

CMRO_2_ is also modulated by body temperature. Hypothermia leads to a dose-dependent reduction in CMRO_2_, thereby lowering CBF [37]. Conversely, hyperthermia raises both CMRO_2_ and CBF, although this relationship is limited to body temperatures below 42 °C; beyond this threshold, CMRO_2_ begins to decline, likely due to cellular injury. Clinically, profound hypothermia is not practically accomplished in our practice and carries risks outside of the scope of this paper.

#### 3.2.4. Anesthetic Pharmacodynamics

Anesthetic agents also exert significant influence on CMRO_2_ and CBF. Propofol, a commonly used intravenous anesthetic for both induction and maintenance in neurosurgical cases, decreases CMRO_2_ and CBF while preserving autoregulatory responses, including PaCO_2_ reactivity [38]. Etomidate similarly reduces CMRO_2_ but carries the risk of adrenal suppression, particularly concerning in prolonged or critically ill cases [39,40]. In contrast, ketamine traditionally has been associated with increased CMRO_2_, ICP, and CBF; however, more recent studies—especially when ketamine is used in multimodal anesthetic regimens alongside agents like propofol—have challenged these assumptions, with some suggesting a potential neuroprotective role [41].

Volatile inhaled anesthetics such as sevoflurane, isoflurane, and desflurane produce a dose-dependent decrease in CMRO_2_, similar to propofol and etomidate. However, at concentrations above 1.0 minimum alveolar concentration (MAC), these agents can increase both ICP and CBF [42]. Additionally, volatile anesthetics have been shown to inhibit both somatosensory evoked potentials (SSEPs) and motor evoked potentials (MEPs) in a dose-dependent fashion [43]. When used at low doses, inhaled anesthetics can be safely incorporated into neurosurgical anesthetic plans, including during EC–IC bypass—without causing detrimental alterations in intracranial dynamics or neuromonitoring signals.

Cerebral perfusion is a multifactorial and dynamic physiological process influenced by variables such as mean arterial pressure MAP, ICP, PaCO_2_, CMRO_2_, and the pharmacologic effects of anesthetic agents. While the complex interplay of these factors presents significant challenges during neurosurgical procedures, a thorough understanding of each offers critical opportunities for targeted neuroanesthetic intervention. By leveraging this knowledge, anesthesiologists can optimize cerebral perfusion and create ideal perioperative conditions that support the success of intricate neurosurgical operations.

## 4. Perioperative Considerations

**Figure 1:**
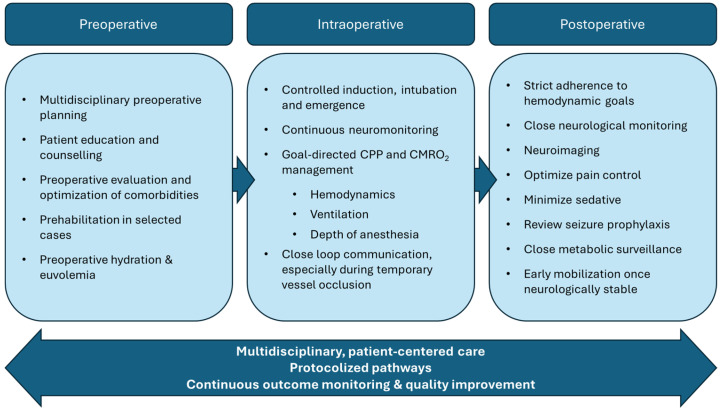
Summary of perioperative considerations for EC–IC bypass.

The perioperative management of patients undergoing EC–IC bypass presents unique challenges that demand a coordinated, multidisciplinary approach. Optimal outcomes depend on seamless integration of preoperative planning, precise intraoperative anesthetic and hemodynamic strategies, and vigilant postoperative monitoring. This is summarized in Figure 1. Each phase of care must be tailored to preserve cerebral perfusion, minimize ischemic risk, and support timely neurological evaluation. An in-depth understanding of cerebrovascular physiology, surgical objectives, and institution-specific protocols is essential to anticipate complications and intervene appropriately. The following sections outline our institution’s approach to perioperative management, emphasizing principles that can be adapted to a variety of clinical settings.

### 4.1. Preoperative Considerations

As with any major surgical procedure, EC–IC bypass surgery demands thorough preoperative planning. For neuroanesthesiologists, key considerations can be broadly categorized into three major domains: patient selection, multidisciplinary planning, and presurgical optimization. Each of these elements plays a critical role in ensuring favorable surgical outcomes and minimizing perioperative risks.

#### 4.1.1. Patient Selection

Anesthesiologists are often limited in their ability to contribute to patient selection for EC–IC bypass, as they typically first encounter the patient on the day of surgery, after a prolonged preoperative evaluation period and the development of a therapeutic alliance with the neurosurgical team. Nevertheless, an understanding of the criteria for appropriate patient selection remains critical for anticipating intraoperative challenges and optimizing perioperative management.

As discussed above, early randomized trials failed to demonstrate a benefit of EC–IC bypass in unselected patients with atherosclerotic cerebrovascular disease; however, more recent studies have suggested a potential benefit in select patients with symptomatic cerebral ischemia and evidence of hemodynamic compromise despite maximal medical therapy [5,44]. Additional accepted indications include complex intracranial aneurysms requiring parent vessel sacrifice, skull base tumors involving major cerebral vessels, and Moyamoya disease with progressive ischemic symptoms refractory to medical management [12,14].

Beyond the primary indication for EC–IC bypass, additional patient-specific factors, including neurological status, age, and medical comorbidities—must be thoroughly evaluated during the preoperative assessment. Younger patients and those with progressive cerebral ischemia or large, surgically challenging aneurysms not amenable to endovascular therapy are generally considered to derive the greatest benefit from bypass procedures [45]. Conversely, individuals with poor functional status or significant systemic comorbidities may be at increased risk for perioperative complications and may not represent optimal surgical candidates.

Establishing a clear preoperative neurological baseline is essential, as it provides a reference point for serial mental status evaluations during the postoperative period and long-term follow-up. Such assessments are critical for early detection of neurological deterioration and guiding subsequent interventions.

Thorough patient counseling is a critical component of preoperative planning for EC–IC bypasses and cannot be overemphasized. Patients typically present for evaluation in the outpatient neurosurgical setting, often exhibiting wide variability in both risk tolerance and expectations regarding surgical outcomes. Early identification and understanding of these individual factors are essential for appropriate patient selection and aligning procedural goals well before the day of surgery.

The informed consent process must include a detailed discussion of the potential risks associated with EC–IC bypass, including perioperative stroke, graft failure, infection, and seizures [44,46]. In addition, patients should be counseled on the anticipated benefits, procedural limitations, and available alternatives, ensuring realistic expectations and facilitating shared decision-making [46].

#### 4.1.2. Multidisciplinary Planning

Effective planning for EC–IC bypass requires close collaboration among neurosurgeons, vascular neurologists, and neuroradiologists. Although a comprehensive discussion of neuroimaging is beyond the scope of this paper, it is a cornerstone of preoperative assessment and surgical planning. Magnetic resonance imaging (MRI) and computed tomography angiography (CTA) are used to delineate cerebrovascular anatomy, identify stenoses or occlusions, and assess collateral circulation. Digital subtraction angiography (DSA) remains the gold standard for detailed vascular mapping. In addition, perfusion imaging—such as CT or MR perfusion—provides quantitative evaluation of CBF, cerebral blood volume (CBV), and mean transit time (MTT), aiding in the identification of regions with hemodynamic compromise [47,48]. Patients exhibiting poor perfusion and impaired autoregulatory capacity within the affected vascular territory are more likely to derive benefit from revascularization.

Mapping of potential donor and recipient vessels is also critical. The superficial temporal artery (STA) is a commonly used donor vessel, and its course, caliber, and accessibility are evaluated preoperatively using duplex ultrasonography or angiographic techniques.

Although neuroanesthesiologists and neurocritical care specialists may not encounter the patient until the day of surgery, early perioperative involvement of these teams is advantageous. Interdisciplinary planning facilitates continuity of care and optimizes outcomes in this complex microsurgical population [49]. Preoperative education regarding the EC–IC procedure, as well as clearly defined intraoperative and postoperative care protocols, allows the large team of care providers the patient will encounter to better anticipate the physiological challenges unique to cerebral revascularization.

#### 4.1.3. Presurgical Optimization

Collaboration with a robust anesthesiology preoperative clinic can be helpful in ensuring a patient is optimized before their EC–IC bypass. The anesthesiologist can provide a preoperative assessment that includes evaluation of airway and venous access, anticipatory education on general anesthesia, and answering questions the patient has about the anesthetic and perioperative plan. Ideally, these clinics involve an anesthesiologist familiar with EC–IC bypass who is also acquainted with the logistics of coordinating care between multiple subspecialities that medically complex patients, like those pending EC–IC bypass, require.

Cardiovascular comorbidities, including coronary artery disease, hypertension, and hyperlipidemia, commonly accompany patients with underlying cerebrovascular atherosclerotic disease and should be well controlled prior to surgery [50]. While there’s a paucity of research surrounding controlling these conditions specifically in EC–IC bypass outcomes, the benefits of optimizing cardiac health preoperative can be extrapolated from other major surgical fields, including vascular surgery [51].

Each medication, including both prescribed and over-the-counter agents, a patient takes must be carefully considered in the perioperative period. Examination of the wide range of possible medication and supplement indications while simultaneously weighing the risks and benefits of continuing or discontinuing these agents, such as perioperative bleeding and thrombotic risk, is critical. Often patients being evaluated for EC–IC bypass are on antiplatelet medications and are continued on these perioperatively. However, the neurosurgeon should be aware of this and make clear antiplatelets hold requirements, with discontinuation time periods that are adequate to minimize perioperative concerns.

There is also an emerging concept in the field of preoperative medicine known as prehabilitation, where patients undergo a combination of physical exercise, psychobehavioral hygiene, and apposite nutrition prior to surgery with the hopes of mitigating a decline in functional status postoperatively [52]. While prehabilitation is an emerging concept in surgery, it is important to consider the specific risks and benefits for patients undergoing EC–IC bypass. Caution is warranted when recommending physical prehabilitation for individuals with acute or decompensated cardiovascular or pulmonary disease, given the elevated risks associated with strenuous activity. However, even patients unable to participate in rigorous physical programs may benefit from non-physical prehabilitation strategies such as macronutrient supplementation, evidence-based nutritional plans, cognitive restructuring, and smoking cessation. These interventions are particularly relevant for the EC–IC bypass population, where optimizing nutritional status and promoting tobacco use reduction can play a critical role in supporting a successful, patient-centered recovery. The duration of prehabilitation is dependent on the case urgency, cases that require urgent intervention like select coronary artery bypasses or certain oncologic surgical interventions should not be delayed solely due to prehabilitation. While the concept of prehabilitation has not yet been studied in EC–IC bypass, we can infer from other major surgical procedures like cardiac surgery that appropriate prehabilitation may benefit patients pending EC–IC bypass [53].

### 4.2. Intraoperative Considerations

The intraoperative anesthetic management for EC–IC bypass is critical for optimizing surgical outcomes by maintaining adequate cerebral perfusion, minimizing complications, and facilitating neurophysiological monitoring. The following are common practices in our experience as a leading EC–IC bypass center and should be considered by anesthesiologists during EC–IC bypass procedures.

#### 4.2.1. Fluid Management

Preoperative and intraoperative volume status must be carefully assessed and managed to optimize hemodynamic stability during EC–IC bypass surgery. Patients typically present in a fasting state, and any resultant volume deficits should be corrected prior to induction of general anesthesia to ensure adequate preload and cardiac output. Maintaining sufficient cardiac output is essential for supporting MAP and, consequently, CPP, particularly during anesthetic induction and periods of temporary vessel occlusion when collateral circulation is relied upon.

The patient’s underlying cardiac function should guide fluid administration strategies. In individuals with normal biventricular function, a pre-induction bolus of isotonic crystalloid is commonly administered to support hemodynamic parameters. In patients with impaired cardiac function or limited volume tolerance, the goal remains to maintain euvolemia but through more nuanced monitoring [54]. This may include interpretation of invasive arterial waveform parameters, including pulse pressure variation; point-of-care laboratory assessments, including serum electrolytes, and lactate; and urine output trends. In such cases, individualized volume management is critical to avoid exacerbating cardiac compromise while ensuring adequate perfusion of vulnerable cerebral territories.

#### 4.2.2. Anesthetic Agent Selection

Anesthetic management for EC–IC bypass must support three principal objectives: hemodynamic stability, preservation of neurophysiologic monitoring fidelity, and timely emergence to allow immediate postoperative neurological assessment. A balanced anesthetic approach utilizing low doses of multiple agents can achieve these goals while minimizing drug-specific adverse effects. Commonly employed regimens include propofol titrated to maintain a Bispectral Index (BIS) between 40–60, sevoflurane at or below 0.5 MAC, dexmedetomidine at 0.2–0.3 mcg/kg/hr, and remifentanil at 0.05–0.08 mcg/kg/min. This multimodal strategy leverages the individual benefits of each agent while reducing the likelihood of dose-dependent complications.

Propofol provides favorable reductions in CMRO_2_ and CBF. However, when used as a sole total intravenous anesthetic (TIVA) agent at higher doses, delayed emergence may occur due to its context-sensitive half-life. Similar delayed recovery may be seen with higher doses of dexmedetomidine [55]. In contrast, remifentanil, which is metabolized by nonspecific plasma esterases, exhibits an extremely short context-sensitive half-life, allowing rapid emergence even after prolonged infusions. Nevertheless, high doses of remifentanil have been associated with postoperative hyperalgesia [56]. As discussed above, sevoflurane and other volatile agents, when administered at higher concentrations, can increase CBF and interfere with neurophysiologic monitoring, thus necessitating careful titration to ≤0.5 MAC. TIVA necessitates consistent and durable venous access throughout the surgical procedure. In our practice, it is standard to establish multiple peripheral intravenous cannulations prior to surgical incision. This approach mitigates the risk associated with potential complications such as intravenous cannula infiltration or the loss of venous access—events that can be particularly problematic when access to alternative sites is restricted due to surgical positioning. These challenges are further compounded in microscopic neurosurgical procedures, where intraoperative repositioning of the patient is markedly limited.

Benzodiazepines, particularly midazolam, are frequently used for premedication or induction in general anesthesia; however, their use is discouraged in EC–IC bypass procedures. At higher doses, midazolam may suppress cortical activity and adversely affect neuromonitoring signals, including somatosensory evoked potentials and electroencephalography [57]. Furthermore, residual sedation may obscure early postoperative neurologic assessment. Similarly, nitrous oxide is avoided due to its known effects on increasing cerebral perfusion, even at concentrations as low as 30% in oxygen-enriched mixtures, and its potential to degrade the quality of neurophysiologic monitoring [58].

Complete neuromuscular paralysis is maintained throughout EC–IC bypass procedures to prevent patient movement, which could have catastrophic consequences in the setting of delicate cerebrovascular surgery performed under the operating microscope. In addition to enhancing surgical safety, paralysis facilitates optimal exposure and manipulation of donor and recipient vessels. Neuromuscular blockade must be carefully titrated and continuously monitored to ensure an appropriate depth of paralysis compatible with both surgical conditions and intraoperative monitoring requirements [59]. In some cases, the neurophysiology team may assist in quantifying neuromuscular blockade using train-of-four (TOF) monitoring or related techniques.

Reversal of neuromuscular blockade at the conclusion of the procedure must be undertaken with particular care. Given the duration of surgery and the use of deep neuromuscular blockade, incomplete reversal may delay emergence and interfere with timely postoperative neurological examination. In our practice, neuromuscular blockade is typically maintained with either intermittent boluses or continuous infusion of rocuronium. At the end of the procedure, generous dosing of sugammadex is employed to ensure rapid and complete reversal, allowing for prompt extubation and a reliable early neurologic assessment in the immediate postoperative period [59].

#### 4.2.3. Hemodynamic Goals

Continuous intraoperative monitoring is essential during EC–IC bypass procedures to ensure patient safety and guide anesthetic and hemodynamic management. Standard monitoring includes all American Society of Anesthesiologists (ASA) recommended parameters, such as electrocardiography, pulse oximetry, capnography, and core temperature assessment. Invasive arterial blood pressure monitoring is established prior to induction to allow for heartbeat-to-heartbeat blood pressure measurement and to facilitate frequent arterial blood gas analysis.

Intraoperative neurophysiologic monitoring is performed by a dedicated neuromonitoring team. SSEPs are routinely employed to assess cortical perfusion, MEP may be used selectively, depending on lesion location, surgical approach, and patient-specific neurological risk factors beyond the scope of this paper.

Maintaining optimal cerebral perfusion is of paramount importance throughout EC–IC bypass [60]. Anesthetic management should aim to preserve blood pressures at levels sufficient to support perfusion through compromised or collateral-dependent vascular territories. In general, we aim to maintain systolic blood pressures within approximately 20% of the patient’s baseline, using vasopressors such as phenylephrine, vasopressin, or norepinephrine as needed [61].

Our practice focuses special attention on two specific phases of the procedure that require distinct hemodynamic target that may result in a departure from the 20% of baseline hemodynamic goal: the extradural and intradural phases. During the extradural phase—including harvesting of the STA —systolic blood pressure is typically maintained between 150–160 mmHg or higher, depending on patient-specific factors, to optimize donor vessel perfusion and reduce the risk of vasospasm. During the intradural phase, particularly during temporary vessel occlusion and microvascular anastomosis, systolic blood pressure is generally reduced to 120–130 mmHg to minimize vessel wall tension during microanastomosis, optimize the surgical field with minimal bleeding, and establish a lower blood pressure post-bypass to reduce hyperperfusion injury, provided that SSEP and electroencephalographic (EEG) signals remain stable. Blood pressure management throughout the case should be dynamic and guided by continuous neurophysiologic feedback to ensure cerebral perfusion is preserved across vulnerable territories.

Ventilatory management also plays a critical role in optimizing cerebral perfusion. Given the cerebrovascular sensitivity to carbon dioxide, arterial PaCO_2_ should be maintained near 40 mmHg. Both hyperventilation and resultant hypocapnia must be avoided, as these can induce cerebral vasoconstriction and reduce perfusion, particularly in already ischemic regions. This is especially important during periods of temporary vessel occlusion, where maximal cerebral blood flow is essential for tissue preservation.

#### 4.2.4. Burst Suppression

Temporary arterial occlusion is a routine component of the intradural phase of EC–IC bypass. During these periods of intentional cerebral ischemia, pharmacologic burst suppression may be employed to reduce cerebral metabolic demand and offer neuroprotection. Burst suppression is an EEG pattern characterized by alternating periods of high-amplitude electrical activity (“bursts”) and isoelectric or low-voltage intervals (“suppressions”). This state reflects a significant reduction in cortical neuronal activity and is associated with a lower CMRO_2_, thereby theoretically increasing ischemic tolerance [62].

Burst suppression is typically achieved using intravenous anesthetic agents, most commonly propofol, administered as boluses or infusions titrated to EEG response. Real-time interpretation by the intraoperative neurophysiology team is critical to confirm adequate suppression and avoid excessive dosing that may delay postoperative emergence.

At our institution, burst suppression is common in high-flow bypass cases or when temporary occlusion involves proximal intracranial vessels, both of which carry increased risk for ischemic injury. In these scenarios, metabolic suppression may mitigate the risk of neuronal damage during transient hypoperfusion.

All arterial occlusion and reperfusion time points must be meticulously recorded to assist with postoperative evaluation. Accurate documentation can facilitate correlation of intraoperative events with the development of new neurological deficits and inform management in the postoperative period.

#### 4.2.5. Emergence

Timely emergence from anesthesia is essential for early neurological evaluation following EC–IC bypass. Changes in neurologic exam immediately post bypass warrant further investigation, including neuroimaging of the head with angiography. When propofol is used in combination with low-dose volatile agents, dexmedetomidine, and remifentanil, a balance can be achieved between providing adequate intraoperative anesthesia and facilitating rapid recovery. Anesthetic depth should be reduced in a controlled manner toward the end of the procedure to prevent abrupt hemodynamic changes and to ensure a smooth transition to the postoperative phase of care.

Just as blood pressure management is essential intraoperatively, it remains a critical component of care in the immediate postoperative period, particularly periextubation. Uncontrolled hypertension during this phase may precipitate serious hemorrhagic complications, especially in patients with impaired cerebrovascular autoregulation due to chronic ischemia [63,64]. Hemorrhage may occur at the anastomotic site, from subdural or epidural venous structures, or as intraparenchymal bleeding related to reperfusion injury [65].

Reperfusion hemorrhage is a recognized complication in patients with longstanding cerebral hypoperfusion. In such individuals, structurally compromised microvasculature and neural tissue may be unable to tolerate the sudden restoration of flow, increasing the risk of vessel rupture and parenchymal damage [66]. This underscores the importance of gradual normalization of cerebral perfusion and meticulous blood pressure control during and after revascularization.

Postoperative blood pressure targets should be clearly established through interdisciplinary collaboration with the neurosurgical team prior to emergence from anesthesia. Clear communication of these parameters ensures continuity of care and enables prompt intervention in the event of hemodynamic instability, thereby reducing the likelihood of pressure-related complications in the early postoperative period.

### 4.3. Postoperative Considerations

Immediately following EC–IC bypass surgery, patients at our institution are admitted directly to a dedicated Neurocritical Care Unit (NCCU), where they receive specialized postoperative management. In this setting, continuous hemodynamic and neurologic monitoring is provided by experienced critical care nurses and neurointensivists, allowing for early identification and intervention in the event of complications such as ischemia, hemorrhage, or hyperperfusion syndrome.

An important consideration following EC–IC bypass is the risk of both cerebral hyperperfusion and ischemic complications, particularly in patients with Moyamoya disease, who are considered high risk [64]. The fragile collateral circulation and chronically hypoperfused cerebral tissue in Moyamoya patients predispose them to cerebral hyperperfusion syndrome postoperatively, which may manifest as transient neurological deficits, seizures, or intracerebral hemorrhage [67]. Conversely, insufficient graft flow or perioperative hypotension can exacerbate ischemia, leading to new infarcts. Meticulous perioperative hemodynamic control, vigilant neurological monitoring, and the use of advanced imaging modalities to assess cerebral perfusion are critical in mitigating these risks and ensuring optimal surgical outcomes.

Neurological assessments are performed frequently—typically every hour—during the initial postoperative period. A comprehensive neurologic examination is documented upon emergence from anesthesia and is directly communicated during the formal handoff to NCCU personnel. This exam establishes a reliable baseline for ongoing serial assessments and guides interventions based on deviations from expected neurologic function. The frequency and scope of these evaluations are determined by institutional NCCU protocols, tailored to each patient’s perioperative risk profile.

Hemodynamic management in the NCCU emphasizes gradual and deliberate control of blood pressure. Continuous arterial pressure monitoring facilitates the titration of short-acting vasoactive agents to maintain systolic and mean arterial pressures within individualized target ranges, avoiding abrupt fluctuations that could jeopardize graft perfusion or precipitate hemorrhage. Blood pressure goals are established collaboratively between neurosurgery and neurocritical care teams prior to emergence and are adjusted postoperatively based on intraoperative events, perfusion imaging, and neuromonitoring data.

Additional elements of postoperative care include the maintenance of optimal intravascular volume status, appropriate electrolyte repletion, implementation of early mobilization protocols, and administration of seizure prophylaxis when clinically indicated [68]. Neuroimaging is routinely obtained in the early postoperative period; a non-contrast head computed tomography (CT) scan is typically performed within 24 hours of surgery to evaluate for postoperative hemorrhage, infarction, and hydrocephalus [69]. The development of hydrocephalus may necessitate shunt placement in the postoperative period [16]. If bypass patency needs to be assessed, CT angiography or digital subtraction angiography may be performed based on clinical or surgical criteria [70].

Postoperative management is guided by interdisciplinary collaboration among neurosurgeons, neurointensivists, neuroanesthesiologists, neurophysiologists, and specialized nursing staff. This collaborative approach is embedded in our institution’s cerebrovascular care pathways and is critical to ensuring a seamless transition from intraoperative management to postoperative recovery and long-term rehabilitation.

## 5. Conclusions

EC–IC bypass is performed for either flow augmentation in patients with occlusive cerebrovascular disease, including Moyamoya disease, or for flow replacement in the treatment of complex pathologies such as giant aneurysms. Anesthetic management in these cases is particularly challenging, with limited guidance available in the literature. Patients with diffuse vascular disease often present with comorbidities that complicate the maintenance of optimal cerebral perfusion. At our institution, close collaboration between neurological surgery and anesthetic teams, supported by a dedicated group of neuroanesthesiologists, has been essential in achieving hemodynamic stability and timely emergence. In the context of limited published data and a high procedural volume of approximately 100 EC–IC bypasses annually at our institution, the use of the above protocol has been associated with a low rate of perioperative ischemic events and facilitation of timely postoperative emergence. We hope that this manuscript provides meaningful insights for other centers treating similar patient populations and future research directions.

## Data Availability

No new data were created or analyzed in this study. Data sharing is not applicable to this article.

## Abbreviations

The following abbreviations are used in this manuscript:

ASA	American Society of Anesthesiologists (classification)
BIS	Bispectral Index
CBF	Cerebral blood flow
CBV	Cerebral blood volume
CMRO_2_	Cerebral metabolic rate of oxygen consumption
CPP	Cerebral perfusion pressure
CSF	Cerebrospinal fluid
CT	Computed tomography
EC–IC	Extracranial–intracranial
ICA	Internal carotid artery
ICP	Intracranial pressure
MAC	Minimum alveolar concentration
MAP	Mean arterial pressure
MCA	Middle cerebral artery
MLCK	Myosin light chain kinase
MMD	Moyamoya disease
MTT	Mean transit time
NCCU	Neurocritical care unit
NO	Nitric oxide
PaCO_2_	Partial pressure of carbon dioxide in cerebral arteries
PaO_2_	Partial pressure of oxygen in cerebral arteries
SSEP	Somatosensory evoked potential
STA	Superficial temporal artery
TIVA	Total intravenous anesthesia

## References

[1] Fisher M. (1951). OCCLUSION OF THE INTERNAL CAROTID ARTERY. Arch. Neurol. Psychiatry.

[2] Yasagril M.G. (1969). Reconstructive and Constructive Surgery of the Cerebral Arteries in Man. Microsurgery applied to neurosurgery. Microsurgery: Applied to Neurosurgery.

[3] Doherty R.J., Caird J., Crimmins D., Kelly P., Murphy S., McGuigan C., Tubridy N., King M.D., Lynch B., Webb D. (2020). Moyamoya disease and moyamoya syndrome in Ireland: Patient demographics, mode of presentation and outcomes of EC–IC bypass surgery. Ir. J. Med Sci. (1971-).

[4] Muroi C., Khan N., Bellut D., Fujioka M., Yonekawa Y. (2011). Extracranial–intracranial bypass in atherosclerotic cerebrovascular disease: Report of a single centre experience. Br. J. Neurosurg..

[5] Brenner L.B., Sousa M.P., Andreão F.F., Prestes M.Z., Palavani L.B., Batista S., Koester S.W., Rabelo N.N., Bertani R., Welling L.C. (2024). Clinical and Technical Outcomes of Intracranial-Intracranial Bypass for Treating Complex Intracranial Aneurysms: An Analysis of 255 Patients. World Neurosurg..

[6] Amin-Hanjani S., Butler W.E., Ogilvy C.S., Carter B.S., Barker F.G. (2005). Extracranial—intracranial bypass in the treatment of occlusive cerebrovascular disease and intracranial aneurysms in the United States between 1992 and 2001: A population-based study. J. Neurosurg..

[7] Awano T., Sakatani K., Yokose N., Kondo Y., Igarashi T., Hoshino T., Nakamura S., Fujiwara N., Murata Y., Katayama Y. (2010). Intraoperative EC–IC Bypass Blood Flow Assessment With Indocyanine Green Angiography in Moyamoya and Non-Moyamoya Ischemic Stroke. World Neurosurg..

[8] Dengler J., Cabraja M., Faust K., Picht T., Kombos T., Vajkoczy P. (2013). Intraoperative neurophysiological monitoring of extracranial-intracranial bypass procedures. J. Neurosurg..

[9] Grüter B.E., Tosic L., Voglis S., Vasella F., Mutschler V., Bichsel O., Scherrer N., Regli L., Esposito G. (2021). Trends in Literature on Cerebral Bypass Surgery: A Systematic Review. Cerebrovasc. Dis..

[10] Meling T.R., Patet G. (2020). The role of EC–IC bypass in ICA blood blister aneurysms—a systematic review. Neurosurg. Rev..

[11] Schaller B. (2008). Extracranial-Intracranial Bypass to Reduce the Risk of Ischemic Stroke in Intracranial Aneurysms of the Anterior Cerebral Circulation: A Systematic Review. J. Stroke Cerebrovasc. Dis..

[12] Burkhardt J.-K., Lawton M.T. (2019). Practice Trends in Intracranial Bypass Surgery in a 21-Year Experience. World Neurosurg..

[13] Sanai N., Zador Z., Lawton M.T. (2009). Bypass surgery for complex brain aneurysms. Neurosurgery.

[14] Wolfswinkel E.M., Landau M.J., Ravina K., Kokot N.C., Russin J.J., Carey J.N. (2018). EC-IC bypass for cerebral revascularization following skull base tumor resection: Current practices and innovations. J. Surg. Oncol..

[15] Wessels L., Hecht N., Vajkoczy P. (2018). Bypass in neurosurgery—indications and techniques. Neurosurg. Rev..

[16] Gazyakan E.M., Lee C.-Y., Wu C.-T., Tsao C.-K., Craft R., Henry S.L., Cheng M.-H.M., Lee S.-T. (2015). Indications and Outcomes of Prophylactic and Therapeutic Extracranial-to-intracranial Arterial Bypass for Cerebral Revascularization. Plast. Reconstr. Surg.–Glob. Open.

[17] Joshi G., Yamada Y., Thavara B.D., Tanaka R., Miyatini K., Nakao K., Kawase T., Takizava K., Kato Y. (2020). EC–IC Bypass; Our experience of cerebral revascularization with intraoperative Dual-Image Video Angiography (Diva). Asian J. Neurosurg..

[18] Matsukawa H., Tanikawa R., Kamiyama H., Tsuboi T., Noda K., Ota N., Miyata S., Tokuda S. (2016). The Valveless Saphenous Vein Graft Technique for EC–IC High-Flow Bypass: Technical Note. World Neurosurg..

[19] Zhang Y., Sia S., Morgan M., Qian Y. (2012). Flow resistance analysis of extracranial-to-intracranial (EC–IC) vein bypass. J. Biomech..

[20] Dubovoy A.V., Ovsyannikov K.S., Guzhin V.E., Cherepanov A.V., Galaktionov D.M., Perfil’eV A.M., Sosnov A.O. (2017). The use of high-flow extracranial-intracranial artery bypass in pathology of the cerebral and brachiocephalic arteries: Technical features and surgical outcomes. Vopr. neirokhirurgii Im. N.N. Burdenko.

[21] Herzig R., Hluštík P., Urbánek K., Vaverka M., Buřval S., Macháč J., Vlachová I., Křupka B., Bártková A., Šaňák D. (2004). Can We Identify Patients With Carotid Occlusion Who Would Benefit From Ec/ic Bypass? Review. Biomed. Pap..

[22] Patel H.C., Mcnamara I.R., Al-Rawi P.G., Kirkpatrick P.J. (2010). Improved cerebrovascular reactivity following low flow EC/IC bypass in patients with occlusive carotid disease. Br. J. Neurosurg..

[23] Sia S.F., Morgan M.K. (2013). High flow extracranial-to-intracranial brain bypass surgery. J. Clin. Neurosci..

[24] Hendrikse J., van der Zwan A., Ramos L.M., van Osch M.J., Golay X., Tulleken C.A., van der Grond J. (2005). Altered Flow Territories after Extracranial-Intracranial Bypass Surgery. Neurosurgery.

[25] Vu E.L., Brown C.H., Brady K.M., Hogue C.W. (2024). Monitoring of cerebral blood flow autoregulation: Physiologic basis, measurement, and clinical implications. Br. J. Anaesth..

[26] Smith M. (2015). Cerebral perfusion pressure. Br. J. Anaesth..

[27] Liu J., Zhu Y.-S., Hill C., Armstrong K., Tarumi T., Hodics T., Hynan L.S., Zhang R. (2013). Cerebral Autoregulation of Blood Velocity and Volumetric Flow During Steady-State Changes in Arterial Pressure. Hypertension.

[28] Rossi J.L., Todd T., Bazan N.G., Belayev L. (2013). Inhibition of Myosin Light-Chain Kinase Attenuates Cerebral Edema after Traumatic Brain Injury in Postnatal Mice. J. Neurotrauma.

[29] White H., Venkatesh B. (2008). Cerebral Perfusion Pressure in Neurotrauma: A Review. Anesthesia Analg..

[30] Lidington D., Wan H., Bolz S.-S. (2021). Cerebral Autoregulation in Subarachnoid Hemorrhage. Front. Neurol..

[31] Iadecola C., Davisson R.L. (2008). Hypertension and Cerebrovascular Dysfunction. Cell Metab..

[32] Ruland S., Aiyagari V. (2007). Cerebral Autoregulation and Blood Pressure Lowering. Hypertension.

[33] Yoon S., Zuccarello M., Rapoport R.M. (2012). pCO_2_ and pH regulation of cerebral blood flow. Front. Physiol..

[34] Yoshihara M., Bandoh K., Marmarou A. (1995). Cerebrovascular carbon dioxide reactivity assessed by intracranial pressure dynamics in severely head injured patients. J. Neurosurg..

[35] Duffin J., Mikulis D.J., Fisher J.A. (2021). Control of Cerebral Blood Flow by Blood Gases. Front. Physiol..

[36] Freeman R.D., Li B. (2016). Neural–metabolic coupling in the central visual pathway. Philos. Trans. R. Soc. B Biol. Sci..

[37] Bao L., Xu F. (2013). Fundamental research progress of mild hypothermia in cerebral protection. SpringerPlus.

[38] Oshima T., Karasawa F., Satoh T. (2002). Effects of propofol on cerebral blood flow and the metabolic rate of oxygen in humans. Acta Anaesthesiol. Scand..

[39] Renou A.M., Vernhiet J., Macrez P., Constant P., Billeerey J., Khadaroo M., Caillé J. (1978). Cerebral blood flow and metabolism during etomidate anaesthesia in man. Br. J. Anaesth..

[40] Hildreth A.N., Mejia V.A., Maxwell R.A., Smith P.W., Dart B.W., Barker D.E. (2008). Adrenal Suppression Following a Single Dose of Etomidate For Rapid Sequence Induction: A Prospective Randomized Study. J. Trauma Inj. Infect. Crit. Care.

[41] Gregers M.C.T., Mikkelsen S., Lindvig K.P., Brøchner A.C. (2020). Ketamine as an Anesthetic for Patients with Acute Brain Injury: A Systematic Review. Neurocritical Care.

[42] Preethi J., Bidkar P.U., Cherian A., Dey A., Srinivasan S., Adinarayanan S., Ramesh A.S. (2019). Comparison of total intravenous anesthesia vs. inhalational anesthesia on brain relaxation, intracranial pressure, and hemodynamics in patients with acute subdural hematoma undergoing emergency craniotomy: A randomized control trial. Eur. J. Trauma Emerg. Surg..

[43] Xiang B., Jiao S., Zhang Y., Wang L., Yao Y., Yuan F., Chen R., Zhou Q. (2021). Effects of desflurane and sevoflurane on somatosensory-evoked and motor-evoked potential monitoring during neurosurgery: A randomized controlled trial. BMC Anesthesiol..

[44] McDowell F., Flamm E.S. (1986). EC/IC Bypass Study. Stroke.

[45] Li X., Li Y., Wang T., Sun X., Lu G., Xu X., Yang R., Luo J., Bai X., Tong X. (2025). Determining the Optimal Age for Extracranial-Intracranial Bypass Surgery: A Post Hoc Analysis of the CMOSS Randomized Trial. Stroke.

[46] Gobble R.M., Hoang H., Jafar J., Adelman M. (2012). Extracranial-intracranial bypass: Resurrection of a nearly extinct operation. J. Vasc. Surg..

[47] Li C., Cao X., Ma Z., Sun X., Hu F., Wang L. (2018). Effect of pre-surgery assessments on the prognosis of patients received extracranial-intracranial bypass surgery. Restor. Neurol. Neurosci..

[48] Kato N., Kan I., Abe Y., Otani K., Narikiyo M., Nagayama G., Nishimura K., Mori R., Kodama T., Ishibashi T. (2021). Visualization of extracranial-intracranial bypass in moyamoya patients using intraoperative three-dimensional digital subtraction angiography with intravenous contrast injection and robotic C-arm: Patient series. J. Neurosurg. Case Lessons.

[49] Anokwute M.C., Preda V., Di Ieva A. (2023). Determining Contemporary Barriers to Effective Multidisciplinary Team Meetings in Neurological Surgery: A Review of the Literature. World Neurosurg..

[50] Wessels L., Hecht N., Vajkoczy P. (2021). Patients receiving extracranial to intracranial bypass for atherosclerotic vessel occlusion today differ significantly from the COSS population. Stroke.

[51] Bush R.L., Zhan H.T., Purcell S.T. (2015). Preoperative optimization of the vascular surgery patient. Vasc. Heal. Risk Manag..

[52] Tew G.A., Caisley K., Danjoux G. (2022). Preoperative exercise training for adults undergoing elective major vascular surgery: A systematic review. PLoS ONE.

[53] Bargnes V., Davidson S., Talbot L., Jin Z., Poppers J., Bergese S.D. (2024). Start Strong, Finish Strong: A Review of Prehabilitation in Cardiac Surgery. Life.

[54] van der Jagt M. (2016). Fluid management of the neurological patient: A concise review. Crit. Care.

[55] Cascella M., Bimonte S., Di Napoli R. (2020). Delayed Emergence from Anesthesia: What We Know and How We Act. Local Reg. Anesthesia.

[56] Santonocito C., Noto A., Crimi C., Sanfilippo F. (2018). Remifentanil-induced postoperative hyperalgesia: Current perspectives on mechanisms and therapeutic strategies. Local Reg. Anesth..

[57] Bithal P. (2014). Anaesthetic considerations for evoked potentials monitoring. J. Neuroanaesth. Crit. Care.

[58] Dashdorj N., Corrie K., Napolitano A., Petersen E., Mahajan R.P., Auer D.P. (2013). Effects of Subanesthetic Dose of Nitrous Oxide on Cerebral Blood Flow and Metabolism. Anesthesiology.

[59] Naguib M., Brull S.J., Kopman A.F., Hunter J.M., Fülesdi B., Arkes H.R., Elstein A., Todd M.M., Johnson K.B. (2018). Consensus Statement on Perioperative Use of Neuromuscular Monitoring. Anesthesia Analg..

[60] Durga P., Kinthala S., Sahu B.P., Panigrahi M.K., Mantha S., Ramachandran G. (2014). Efficacy and outcomes of perioperative anesthetic management of extracranial to intracranial bypass for complex intracranial aneurysm in the absence of advanced neurological monitoring. J. Anaesthesiol. Clin. Pharmacol..

[61] Lonjaret L., Lairez O., Geeraerts T., Minville V. (2014). Optimal perioperative management of arterial blood pressure. Integr. Blood Press. Control..

[62] Michenfelder J.D. (1974). The Interdependency of Cerebral Functional and Metabolic Effects Following Massive Doses of Thiopental in the Dog. Anesthesiology.

[63] Chui J., Manninen P., Sacho R.H., Venkatraghavan L. (2015). Anesthetic Management of Patients Undergoing Intracranial Bypass Procedures. Anesthesia Analg..

[64] Yu J., Zhang J., Li J., Zhang J., Chen J. (2020). Cerebral Hyperperfusion Syndrome After Revascularization Surgery in Patients with Moyamoya Disease: Systematic Review and Meta-Analysis. World Neurosurg..

[65] I Stiver S., Ogilvy C.S. (2002). Acute hyperperfusion syndrome complicating EC–IC bypass. J. Neurol. Neurosurg. Psychiatry.

[66] Teo K., Choy D.K., Lwin S., Ning C., Yeo T.T., Shen L., Chong V.F., Teoh H.L., Seet R.C., Chan B.P. (2013). Cerebral Hyperperfusion Syndrome After Superficial Temporal Artery-middle Cerebral Artery Bypass for Severe Intracranial Steno-occlusive Disease. Neurosurgery.

[67] Fujimura M., Ito M., Uchino H., Kawabori M., Sugiyama T. (2025). Efficacy and Safety of Combined Revascularization Surgery for Moyamoya Disease: Standard Procedure and Perioperative Management. Trends Treat. Cerebrovasc. Dis..

[68] Nobles K., Cunningham K., Fecondo B., Closs S.M., Donovan K., Kumar M.A. (2024). Mobilization in Neurocritical Care: Challenges and Opportunities. Curr. Neurol. Neurosci. Rep..

[69] Schubert G.A., Biermann P., Weiss C., Seiz M., Vajkoczy P., Schmiedek P., Thomé C. (2014). Risk Profile In Extracranial/Intracranial Bypass Surgery—The Role of Antiplatelet Agents, Disease Pathology, and Surgical Technique In 168 Direct Revascularization Procedures. World Neurosurg..

[70] Hurth H., Hauser T.-K., Haas P., Wang S., Mengel A., Tatagiba M., Ernemann U., Khan N., Roder C. (2021). Early Post-operative CT-Angiography Imaging After EC–IC Bypass Surgery in Moyamoya Patients. Front. Neurol..

